# Retail chicken giblets contaminated with extended-spectrum cephalosporin- and carbapenem-resistant *Salmonella enterica* carrying *bla*CMY-2

**DOI:** 10.14202/vetworld.2022.1297-1304

**Published:** 2022-05-25

**Authors:** Fatma Abdel-Kader, Eman Hamza, Khaled A. Abdel-Moein, Maha A. Sabry

**Affiliations:** Department of Zoonoses, Faculty of Veterinary Medicine, Cairo University, Cairo, Egypt

**Keywords:** carbapenems, extended-spectrum eeeeeeee-lactamase, extended-spectrum cephalosporin-resistant, plasmid AmpC, *Salmonella enterica*

## Abstract

**Background and Aim:**

Chickens are considered as the main source of *Salmonella*, with infection potentially spreading to the public through outlets. The study aimed to investigate poultry shops for *Salmonella enterica* resistant to extended-spectrum cephalosporins-resistant (ESCR) and carbapenems-resistant (CR).

**Materials and Methods:**

Samples were collected from chicken giblets, water tanks, and workers at retail shops. *Salmonella* was isolated and serotyped; the presence of *invA*, *stn*, *ompA*, and *ompF* was determined using polymerase chain reaction (PCR). The isolates were tested for ESCR and CR by a disk-diffusion test; a confirmatory extended-spectrum β-lactamase (ESBL) test was performed by combinational disk-diffusion test with clavulanic acid. The resistant isolates were screened for ESBL (*bla*TEM, *bla*SHV, *bla*CTX-M, and *bla*OXA-1), AmpC *bla*CMY-2, and carbapenemase (*bla*KPC, *bla*NDM, and *bla*OXA-48) genes using PCR.

**Results:**

*S. enterica* was isolated from chicken giblets (13/129) and the 13 isolates were ESCR. Based on the confirmatory ESBL test and CR, the 13 isolates were classified into the following resistance phenotypes: ESBL-producing and CR (n=4), ESBL-producing (n=1), non-ESBL-producing and CR (n=6), and non-ESBL-producing (n=2). All the five isolates with ESBL-producing phenotype carried predominantly *bla*TEM, *bla*SHV, and *bla*CMY-2. Regardless of being phenotypically CR, none of these isolates carried any of the tested carbapenemase genes. Surprisingly, the isolates with non-ESBL phenotype were found to carry *bla*TEM, *bla*SHV, and *bla*CMY-2. The *bla*KPC was present mainly in the isolates with non-ESBL and CR phenotypes. Interestingly, two isolates of the non-ESBL and CR phenotype showed resistance to cefepime, the fourth generation cephalosporins. *Salmonella* was also recovered from the water tanks (2/7) and the workers (2/16). The four isolates were ESCR and showed a non-ESBL-producing and CR phenotype; they harbored *bla*TEM, *bla*SHV, *bla*OXA-1, and *bla*KPC. The *bla*CMY-2 was found in one isolate from water and one from humans. All *Salmonella* isolates carried *invA*, *stn*, *ompA*, and *ompF*.

**Conclusion:**

Virulent ESCR *S. enterica* were identified in retail shops. The isolates carried *bla*CMY-2 and ESBL-genes, with a high proportion showing CR. Transmission of such strains to humans through food leads us to recommend regular inspection of retail outlets for antibiotic-resistant bacteria.

## Introduction

Antibiotic resistance (AR) has greatly increased in many species of bacteria worldwide [1]. In particular, the extended-spectrum cephalosporins-resistant (ESCR) [2] and carbapenem-resistant (CR) bacteria are categorized as critically important [1]. In humans, extended-spectrum cephalosporins (CEPH) are used to treat a broad spectrum of bacterial infections found in the respiratory, skin, and urinary tract [3]. Furthermore, carbapenems are currently the last-line antibiotic group, used against multidrug-resistant Gram-negative bacteria [4].

ESCR are defined as resistant to one or more of the third generation-CEPH [1,2], which are mediated primarily by the extended-spectrum β-lactamases (ESBL) and the AmpC-β-lactamases [5,6]. The most important representatives of ESBL are the three β-lactamases TEM, SHV, and CTX-M [7]. Bacteria over-expressing the AmpC-β-lactamases confer resistance to all β-lactam antibiotics except the fourth generation CEPH and carbapenems [4-6]. In contrast to ESBL, the AmpC-β-lactamases are not inhibited by clavulanic acid and other-β-lactamase inhibitors [5,6]. The AmpC-β-lactamases can be encoded by genes located on chromosomes or plasmids [5,8]. In *Salmonella*, resistance to extended-spectrum CEPH commonly results from the action of the plasmid AmpC-β-lactamase CMY-2 [8-10]. The new definition of CR is resistance to at least one carbapenem drug or production of carbapenemases [1,2]. Carbapenemases can hydrolyze all β-lactam antibiotics, including carbapenems and fourth-generation CEPH [6]. Carbapenemases are a diverse group of β*-*lactamases, the most remarkable of which are the big five enzymes KPC, NDM, OXA-48, IMP, and VIM [7,11]. Interestingly, if the presence of ESBL and AmpC genes is combined with the loss of outer membrane porin, this can confer resistance to carbapenems [12].

Animals, mainly poultry, have been associated with the spread of AR bacteria to humans, with consequences for food safety [13,14]. In recent years, ESCR have been reported among *Salmonella* *enterica* [8-10], which results from the extensive use of ESC in broilers [14]. Although carbapenems are not used to treat *Salmonella*, some studies have shown the emergence of CR *Salmonella* isolated from humans [15-17] and chickens [18]. There is increasing evidence of the spread of AR bacteria through food at retail establishments [8,9,14,19].

The study aimed to investigate poultry shops for *S. enterica* resistant to ESCR and CR. Samples were collected from chicken giblets, one of the cheapest and favorite types of meat, as well as from water tanks used for cleaning chicken carcasses, and workers at the retail shops. This will shed light on the presence and distribution of AR-*Salmonella* in retail chicken products and the possible dissemination to the surrounding environment.

## Materials and Methods

### Ethical approval and Informed consent

Protocols for collection of animal samples and the used methods were performed in accordance with the guidelines of the Institutional Animal Care and Use Committee (IACUC; Number: VetCu01102020205) of the Faculty of Veterinary Medicine, Cairo University, Egypt. The work doesn’t contain clinical studies, samples collected from humans were hand swabs. All participants agreed to participate, and their identity data were omitted from the study.

### Study period and location

The study was conducted from October 2017 to October 2018. Poultry retail shops were randomly selected from different localities in Giza Governorate, Egypt. The samples were processed at Department of Zoonoses, Faculty of Veterinary Medicine, Cairo University, Cairo, Egypt.

### Sample collection

Giblets (neck, liver, and gizzards) were collected from 129 chickens and placed in sterile plastic bags. Water samples were collected from tanks (n=7) used to clean the chicken carcasses and placed in sterile plastic tubes. In addition, hand swab samples were collected from staff (n=16) working at the chicken retail shops who agreed to participate in the study. All samples were transported to the laboratory in an icebox (4°C).

### Isolation, identification, and serotyping of *Salmonella* spp.

Following the International Organization for Standardization [20], the samples were processed and cultured. The chicken giblets were homogenized separately, and 10 g were inoculated into 9 mL sterile buffered peptone water (BPW; Oxoid, Hampshire, UK). In addition, the water samples were filtered through 0.4 μm pore size nitrocellulose filters (Sartorius, Aubagne, France). Each filter was then placed in a tube containing 9 mL BPW [21]. Furthermore, the human hand swab samples were also pre-enriched in sterile BPW. All the BPW-pre-enriched samples were incubated at 37°C for 18 h. Then, 100 µL of the incubated sample was inoculated into 10 mL of the enrichment Rappaport Vassilliadis broth (Oxoid) and further incubated at 42°C for 24 h. A loopful of this incubated broth was cultured on Xylose Lysine Desoxycholate agar (Oxoid) at 37°C for 24–48 h. The growing colonies were subjected to Gram-stain films and biochemical identification using RapID ONE kit (Remel, USA). The confirmed *Salmonella* isolates were serotyped using slide agglutination tests with known polyvalent somatic and flagellar antisera according to the Kauffmann-White scheme [22]. This was done in a reference laboratory for veterinary quality control on poultry production, Animal Health Research Institute, Dokki, Giza, Egypt.

### Antibiotic susceptibility testing

The *Salmonella* isolates were tested for susceptibility to CEPH (cefoxitin [FOX, 10 µg], cefpodoxime [CPD, 10 µg], cefotaxime [CTX, 30 µg], ceftazidime [CAZ, 30 µg], ceftriaxone [CRO, 30 µg], cefepime [CPM, 10 µg]); aztreonam (ATM, 30 µg), and carbapenems (meropenem, 10 µg, ertapenem, 10 µg). The test was carried out using the disk-diffusion method on Mueller–Hinton agar using antibiotic disks (Oxoid Ltd., Healthy Family Co, Cairo, Egypt). A confirmatory ESBL test was performed by the double disk-diffusion test using CTX and CAZ in combination with clavulanic acid (CTX/clavulanic acid, 30/10 µg [CTC]; and CAZ/clavulanic acid, 30/10 µg [CAC]). The results were interpreted according to the guidelines of the Clinical and Laboratory Standards Institute (CLSI) [2].

The resistance to the different groups of antibiotics was defined according to Centers for Disease Control and Prevention [1], and CLSI [2]. The isolates were determined as ESCR, if resistance was observed to at least one of the following CEPH; FOX, CPM, CPD, CAZ, CTX, or CRO. Strains resistant to these CEPH and showed a >5-mm increase in a zone diameter of the combined disk (CAC) or (CTC) or both as compared to the antibiotic alone were considered phenotypically ESBL-producing strains; otherwise, the strains were considered phenotypically non-ESBL-producing strains. The CR was defined as resistance to at least one of the carbapenems or carrying carbapenemase genes.

### Molecular detection of the virulence and outer membrane genes

DNA was extracted from the *Salmonella* isolates using the boiling method [23]. The extracted DNAs were examined for the presence of the virulence genes *invA* [24] and *stn* [25] as well as the major outer membrane protein gene *ompA* [26] and the outer membrane porin gene *ompF* [27]. Uniplex polymerase chain reaction (PCR) was carried out using specific oligonucleotide primers outlined in Table-1 [24-27]. PCR reaction mixtures of 25 µL total volume contain 12.5 µL of 2× Emerald Amp GT PCR master mix (Takara), 4.5 µL water, 6 µL template DNA from each isolate, 1 µL from each primer with a concentration of 20 pmol. Negative control was included containing all components of the PCR mixture but with water instead of template DNA. The PCR reaction mixtures were amplified using thermal profile conditions described in Table-1.

**Table 1:** Sequence of oligonucleotide primers used for PCR amplification of virulence, outer membrane protein and porin, and antibiotic-resistance genes.

Genes	Primer sequence (5’-3’)	Product (bp)	PCR amplification conditions	References
*InvA*	GTGAAATTATCGCCACGTTCGGGCAA TCATCGCACCGTCAAAGGAAGGAACC	284	Initial denaturation at 95°C for 5 min; 35 cycles of 94°C for 30 s, 64°C for 30 s, and 72°C for 45 s: final extension at 72°C for 10 min	[24]
*Stn*	TTGTGTCGCTATCACTGGCAACC ATTCGTAACCCGCTCTCGTCC	617	Initial denaturation at 95°C for 3 min; 25 cycles of 94°C for 1 min, 59°C for 1 min, and 72°C for 1 min; final extension at 72°C for 10 min	[25]
*OmpA*	AGTCGAGCTCATGAAAAAGACAGCTATCGC AGTCAAGCT TTTAAGCCTGCGGCTGAG TTA	1052	Initial denaturation at 94°C for 5 min; 35 cycles of 94°C for 30 s, 55°C for 40 s, and 72°C for 1 min; final extension at 72°C for 10 min	[26]
*OmpF*	CCTGGCAGCGGTGATCC TGGTGTAACCTACGCCATC	519	Initial denaturation at 94°C for 5 min; 35 cycles of 94°C for 30 s, 60°C for 40 s, and 72°C for 45 s; final extension at 72°C for 10 min	[27]
*bla* TEM	CGCCGCATACACTATTCTCAGAATGA ACGCTCACCGGCTCCAGATTTAT	445	Initial denaturation at 95°C for 15 min; 30 cycles of 94°C for 30 s, 62°C for 90 s, and 72°C for 60 s; final extension at 72°C for 10 min	[28]
*bla* SHV	CTTTATCGGCCCTCACTCAA AGGTGCTCATCATGGGAAAG	237		
*bla* CTX-M	ATGTGCAGYACCAGTAARGTKATGGC TGGGTRAARTARGTSACCAGAAYC AGC GG	593		
*bla* OXA-1	ACA CAA TAC ATA TCA ACT TCG C AGT GTG TTT AGA ATG GTG ATC	813		
*bla* CMY-2	AGCGATCCGGTCACGAAATA CCCGTTTTATG CACCCATGA	695	Initial denaturation at 94°Cfor 5 min; 30 cycles of 94°C for 1 min, 61°C for 1 min, and 72°C for 1 min; final extension at 72°C for 5 min	[29]
*bla* KPC	ATG TCA CTG TAT CGC CGT CT TTT TCA GAG CCT TAC TGC CC	882	Initial denaturation at 95°C for 15 min; 30 cycles of 94°C for 1 min, 55°C for 1 min, and 72°C for 2 min; final extension at 72°C for 10 min	[30]
*bla* OXA-48	TTG GTG GCA TCG ATT ATC GG GAG CAC TTC TTT TGT GAT GGC	743		
*bla* NDM	GGT TTG GCG ATC TGG TTT TC CGG AAT GGC TCA TCA CGA TC	621		

PCR=Polymerase chain reaction

All PCR products were subjected to electrophoresis on a 1.5% agarose gel, and a DNA ladder was run simultaneously. Two types of DNA ladder were used depending on the size of the PCR products, Gene ruler 100 bp (100-1000, Fermentas, Malaysia); Gene pilot 100 bp plus (100-1500, Qiagen, USA).

### Molecular detection of resistance-determinant genes

**Figure-1:**
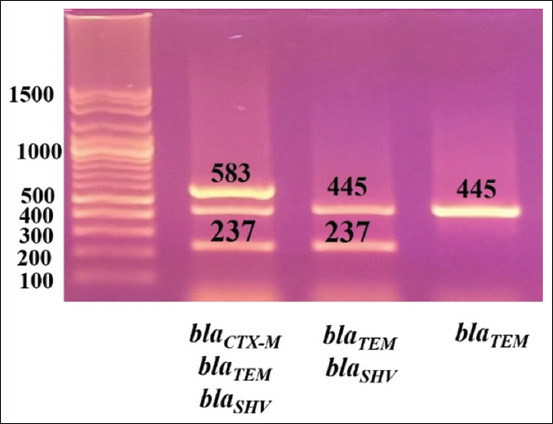
Gel photo of amplified PCR products: *bla*CTX-M (583 bp), *bla*SHV (237 bp), and *bla*TEM (445 bp). Gene pilot 100 bp plus (100-1500).

**Figure-2:**
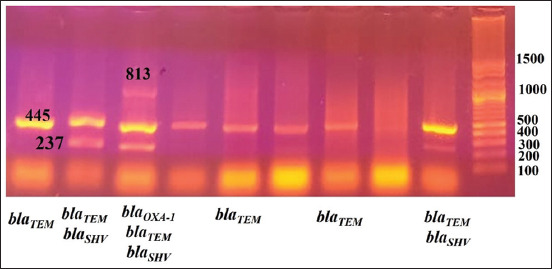
Gel photo of amplified PCR products: *bla*OXA-1 (813 bp), *bla*CTX-M (583 bp), *bla*SHV (237 bp), *bla*TEM (445 bp). Gene pilot 100 bp plus (100-1500).

The extracted DNA was examined for the presence of ESBLs-encoding genes (*bla*CTX-M, *bla*SHV, *bla*TEM, and *bla*OXA-1) by multiplex PCR using specific oligonucleotide primers (Table-1) [28]. The PCR reaction mixtures were prepared from 12.5 µL Emerald Amp GT PCR master mix (Takara Bio Inc., Shiga, Japan), 0.5 µL from each primer with a concentration of 10 pmol, 3 µL template DNA from each isolate, and 5.5 µL water to reach a total volume of 25 µL. The PCR reactions were amplified under the conditions in Table-1; then, the PCR products were electrophoresed on a 1.5% agarose gel (Figures-1 and 2).

Uniplex PCR was used to identify the presence of *bla*CMY-2, the PCR reaction mixtures of 25 µL total volume containing 12.5 µL of 2× Emerald Amp GT PCR master mix (Takara Bio Inc.), 8.5 µL water, 3 µL template DNA from each isolate, and 0.5 µL from each primer with a concentration of 20 pmol. The oligonucleotide primers used for amplification of *bla*CMY-2 and the PCR amplification thermal conditions are found in Table-1 [29]. The PCR product was electrophoresed on a 1% agarose gel to determine the size of the product.

For detection of the carbapenemase-encoding genes (*bla*KPC, *bla*OXA-48, and *bla*NDM), a multiplex PCR was performed using specific oligonucleotide primers (Table-1). All PCR mixtures were prepared from 12.5 µL Emerald Amp GT PCR master mix 2× (Takara, Japan), 0.5 µL from each primer with a concentration of 10 pmol, 3 µL template DNA from each isolate, and 6.5 µL water to reach a total volume of 25 µL. All reactions were amplified using the thermal conditions as given in Table-1 [30]; the PCR products were electrophoresed on 1.5% agarose gel.

Negative control was included in all reactions, which contained all the components of the PCR mixture, but with water instead of the template DNA. In addition, a 100 bp DNA ladder (Size range: 100-1000 bp, Jenna Bioscience GmbH, Jenna, Germany) was run simultaneously to detect the size of the bands.

## Results

*S. enterica* was isolated from 13 samples of the collected 129 chicken giblets. The 13 isolates were of different serotypes and were all positive for *invA*, *stn*, *ompA*, and *ompF* (Table-2). As presented in Table-3, the 13 isolates were resistant to more than one of the tested CEPH. Based on the double-disk-diffusion test with clavulanic acid, five isolates were phenotypically ESBL-producing, and eight were phenotypically non-ESBL-producing.

**Table 2:** Number and serotype characteristics of *Salmonella* isolated from chicken giblets.

Numbers of Examined/Positive samples	Serotypes (numbers) of the isolates	*Virulence (invA)*/Enterotoxin (*stn*)	Outer membrane *OmpA/OmpF*
129/13	*Salmonella* Bargny (n=3),	Positive/Positive	Positive/Positive
	*Salmonella* Kentucky (n=2),		
	*Salmonella* Virchow (n=2),		
	*Salmonella* Enteritidis (n=1),		
	*Salmonella* Typhimurium (n=1),		
	*Salmonella* Infantis (n=1),		
	*Salmonella* Abo (n=2),		
	*Salmonella* Irumu (n=1)		

**Table 3:** Resistance phenotypes and genotypes of the 13 *Salmonella enterica* strains isolated from chicken giblets.

*Salmonella* serotypes	Resistance phenotypes Antibiotics to which the isolates were resistant	Resistance genes
*Salmonella* Kentucky	ESBL-producing and CR CEPH (CPD, CTX, CAZ, CRO) and CR (ETP, MEM)	*bla* TEM,*bla* CMY-2
*Salmonella* Virchow	ESBL-producing* and CR CEPH (FOX, CPD, CTX, CAZ) and CR (ETP)	*bla* SHV,*bla* TEM,*bla* CMY-2
*Salmonella* Typhimurium	ESBL-producing* and CR CEPH (FOX, CPD, CTX, CAZ) and CR (ETP)	*bla* SHV,*bla* TEM,*bla* OXA-1,*bla* CMY-2
*Salmonella* Irumu	ESBL-producing* and CR CEPH (CPD, CTX, CAZ); ATM; and CR (ETP, MEM)	*bla* SHV,*bla* TEM,*bla* CMY-2
*Salmonella* Enteritidis	ESBL-producing* CEPH (FOX, CPD, CTX)	*bla* SHV,*bla* TEM,*bla* CMY-2
*Salmonella* Kentucky	non-ESBL-producing* CEPH (CPD, CTX, CAZ, CRO); CTC*, CAC*; ATM	*bla* TEM,*bla* CMY-2
*Salmonella* Virchow	non-ESBL-producing* and CR CEPH (CPD, CAZ, CRO); CAC*; and CR (ETP)	*bla* SHV,*bla* TEM,*bla* KPC,*bla* CMY-2
*Salmonella* Bargny	non-ESBL-producing* and CR CEPH (CPD, CTX, CRO); CTC*; ATM; and CR (ETP, MEM)	*bla* SHV,*bla* TEM,*bla* OXA-1,*bla* KPC,*bla* CMY-2
*Salmonella* Bargny	non-ESBL-producing* and CR CEPH (CPD, CTX, CRO); CTC*; ATM; and CR (ETP, MEM)	*bla* SHV,*bla* TEM,*bla* OXA-1,*bla* KPC,*bla* CMY-2
*Salmonella* Bargny	non-ESBL-producing* CEPH (CTX, CRO); CTC*	*bla* SHV,*bla* TEM,*bla* CMY-2
*Salmonella* Infantis	non-ESBL-producing* and CR CEPH (CPD, CTX, CRO); CTC; ATM; and CR (ETP, MEM)	*bla* SHV,*bla* TEM,*bla* KPC,*bla* CMY-2
*Salmonella* Abo	non-ESBL-producing* and CR CEPH (CPD, CTX, CAZ, CRO, CPM); CTC*, CAC*; ATM, and CR (ETP)	*bla* SHV,*bla* CTXM,*bla* KPC,*bla* CMY-2
*Salmonella* Abo	non-ESBL-producing* and CR CEPH (CPD, CTX, CAZ, CRO, CPM); CTC*, CAC*; ATM; and CR (ETP, MEM)	*bla* SHV,*bla* CTXM

* The phenotype of the isolates was determined to be ESBL-producing or non-ESBL-producing based on the results of the double disk-diffusion test with clavulanic acid (CTC or CAC), ESBL=Extended-spectrum β-lactamase, ESCR=Extended-spectrum cephalosporins-resistant, CR=Carbapenems-resistant, FOX=Cefoxitin, CPD=Cefpodoxime, CTX=Cefotaxime, CAZ=Ceftazidime, CRO=Ceftriaxone, CPM=Cefepime, ATM=Aztreonam, MEM=Meropenem, ETP=Ertapenem

Of the five phenotypically ESBL-producing strains, four showed resistance to carbapenems, and one was carbapenem-susceptible. The resistance genotype was similar between the CR and the carbapenem-susceptible strains. In this regard, the five strains carried at least one of the ESBL-genes (*bla*SHV, *bla*TEM, and *bla*OXA-1), and the AmpC β-lactamase gene (*bla*CMY-2) but were negative to the examined carbapenemase-genes (Table-3).

Of the eight phenotypically non-ESBL-producing strains, six showed resistance to carbapenems, and two were carbapenem-susceptible. The eight strains carried predominantly at least one of the ESBL-genes (*bla*SHV, *bla*TEM, *bla*OXA-1, and *bla*CTX-M) and *bla*CMY-2, while the carbapenemase *bla*KPC gene was found only in the CR strains. Interestingly, resistance to the 4^th^ generation CPM was found in two isolates that were phenotypically non-ESBL-producing and CR, those isolates carried mainly *bla*SHV and *bla*CTX-M with or without *bla*CMY-2 and *bla*KPC (Table-3).

*Salmonella* was isolated from two of the seven tested water tanks used for cleaning the chicken carcasses (Table-4). The serotypes of the two isolates were Bardo and Magherfelt and carried *invA*, *stn*, *ompA*, and *ompF*. Both isolates were ESCR resistant to more than two of the tested CEPH and were phenotypically non-ESBL-producing and CR. They carried *bla*SHV, *bla*TEM, *bla*OXA-1, and *bla*KPC, while *bla*CMY-2 was found only in the *S*. Bardo isolate.

**Table 4:** Number and antibiotic resistance phenotypes and genotypes of *Salmonella enterica* strains isolated from water tanks used for cleaning the chicken carcasses.

Numbers of Examined/Positive samples	Serotypes/*invA/stn/ompA/ompF*	Resistance phenotypes Antibiotics to which the isolates were resistant	Resistance genes
7/2	*Salmonella* Bardo/positive/positive/positive/positive	non-ESBL-producing* and CR CEPH (CPD, CAZ, CRO); CAC*; and CR (ETP, MEM)	*bla* SHV,*bla* TEM,*bla* OXA-1,*bla* KPC,*bla* CMY-2
	*Salmonella* Magherfelt/positive/positive/positive/positive	non-ESBL-producing* and CR CEPH (FOX, CPD, CTX, CAZ, CRO); CTC*, CAC*; ATM; and CR (ETP, MEM)	*bla* SHV,*bla* TEM,*bla* OXA-1,*bla* KPC

* The phenotype of the isolates was determined to be ESBL-producing or non-ESBL-producing based on the results of the double disk-diffusion test with clavulanic acid (CTC or CAC), FOX=Cefoxitin, CPD=Cefpodoxime, CTX=Cefotaxime, CAZ=Ceftazidime, CRO=Ceftriaxone, CPM=Cefepime, ATM=Aztreonam, MEM=Meropenem, ETP=Ertapenem, ESBL=Extended spectrum β-lactamase, ESCR=Extended-spectrum cephalosporins-resistant, CR=Carbapenems-resistant

*Salmonella* was also isolated from two of the 16 workers involved in handling the chicken carcasses (Table-5). The two isolates were of the serotypes Cyprus and Lindenburg and harbored *invA*, *stn*, *ompA*, and *ompF*. Like the water isolates, the two isolates from humans were phenotypically non-ESBL-producing and CR. They harbored *bla*SHV, *bla*TEM, *bla*OXA-1, and *bla*KPC, whereas only *Salmonella* Cyprus isolate carried *bla*CMY-2.

**Table 5:** Number and antibiotic resistance phenotypes and genotypes of *Salmonella enterica* strains isolated from workers at the retail poultry shops.

Numbers of Examined/Positive samples	Serotypes/*invA/stn/ompA/ompF*	Resistance phenotypes Antibiotics to which the isolates were resistant	Resistance genes
16/2	*Salmonella* Cyprus/positive/positive/positive/positive	non-ESBL-producing* and CR CEPH (CPD, CAZ, CRO); CAC*; and CR (ETP, MEM)	*bla* SHV,*bla* TEM,*bla* OXA-1,*bla* KPC,*bla* CMY-2
	*Salmonella* Lindenburg/positive/positive/positive/positive	non-ESBL-producing* and CR CEPH (CPD, CTX, CRO); CTC*, and CR (ETP, MEM)	*bla* SHV,*bla* TEM,*bla* OXA-1,*bla* KPC

* The phenotype of the isolates was determined to be ESBL-producing or non-ESBL-producing based on the results of the double disk-diffusion test with clavulanic acid (CTC or CAC), FOX=Cefoxitin, CPD=Cefpodoxime, CTX=Cefotaxime, CAZ=Ceftazidime, CRO=Ceftriaxone, CPM=Cefepime, ATM=Aztreonam, MEM=Meropenem, ETP=Ertapenem, ESBL=Extende-spectrum β-lactamase, ESCR=Extended-spectrum cephalosporins-resistant, CR=Carbapenems-resistant

## Discussion

The present study investigated the occurrence of ESCR, ESBL, and CR *Salmonella* at various retail poultry shops. Samples were collected from chicken giblets, water tanks used for cleaning the chicken carcasses, and staff working at the poultry shops. *Salmonella* was isolated from 13 of the 129 giblet samples; the 13 isolates were *invA*, *stn*, *ompA*, and *ompF* positive. In addition, these isolates showed variations in the serotypes, encompassing Kentucky, Enteritidis, Typhimurium, Barny, Virschow, Abo, Irumu, and Infantis. This agrees with a previous study that isolated *Salmonella* having similar serotypes and carrying *invA* and *stn* as well as *ompA* and *ompF* from chickens [31,32]. The 13 current isolates were ESCR, as they exhibited resistance to more than one of the tested CEPH [1,2]. Only five of these 13 isolates were phenotypically ESBL-producing, as the resistance to CEPH was inhibited by the addition of clavulanic acid, while eight isolates were phenotypically non-ESBL-producing, as the addition of clavulanic acid could not inhibit the resistance to CEPH [1,2]. Moreover, of the 13 isolates, ten were phenotypically CR (four of the phenotypically ESBL-producing and six of the phenotypically non-ESBL-producing). Strikingly, both the phenotypically ESBL- and non-ESBL- producing isolates harbored ESBL genes, predominantly *bla*SHV and *bla*TEM, and the pAmpC *bla*CMY-2 genes. This case was previously reported by other studies [33,34], who were interested to know the resistance phenotype of non-ESBL-producing strains. In our opinion, this may be because the presence of ESBL genes does not mean that they are functioning or even expressed, and the resistance to CEPH may be mediated by other genes which are resistant to clavulanic acid, like the *bla*CMY-2 [5,6]. Another reason could be the presence of carbapenemases with ESBLs in these isolates. Indeed, we found that the phenotypically ESBL-producing CR isolates did not carry any of the tested carbapenemase genes. In contrast, the phenotypically non-ESBL-producing CR isolates harbored the carbapenemase *bla*KPC. Carbapenemases can inhibit the action of clavulanic acid and overcome the function of ESBLs [6,7,11]. This finding reveals the importance of combining the analysis of resistance phenotype and genotype when testing for AR. Taken together, the 13 giblet *Salmonella* isolates are ESCR, with the resistance potentially mediated by different mechanisms, including ESBL production and or the cephalosporinase *bla*CMY-2. This agrees with the findings that ESBL-producing *Salmonella* were found in fecal samples from chickens in Egypt [32] and avian isolates from Algeria [28]. Other studies also showed the presence of ESCR *Salmonella* in retail chicken meat, with the resistance mediated by *bla*CMY-2 [8,10].

Our finding of CR *Salmonella* strains in chicken giblets disagrees with studies reporting that such *Salmonella* strains are found in humans but not in food-producing animals [15,16]. This is because carbapenems are used in human medicine but not in veterinary [35]. The first reporting of ESBL- and carbapenemase-producing *Salmonella* was S. Cubana isolated from fecal samples of a 4-year-old child in the USA in 1998 [17]. However, our results concur with a study that showed the emergence of CR *Salmonella* in chickens [18]. Our group has also previously demonstrated the presence of CR in *Klebsiella pneumoniae* isolated from chickens, mediated mainly by *bla*NDM with or without *bla*KPC and *bla*OXA-48 [35]. In the present study, we could find only *bla*KPC, the other genes *bla*NDM and *bla*OXA-48 were absent. Only five of the ten phenotypically CR isolates contained *bla*KPC, indicating that the CR is mediated by this carbapenemase. However, in the other five phenotypically CR isolates, the CR resistance might be mediated by carbapenemases that were not tested in the present study or by mechanisms other than the carbapenemases. One of these mechanisms could be the presence of ESBL genes and *bla*CMY-2, combined with the loss of outer membrane porins (omp) [11,12]. Unfortunately, our results could not confirm this hypothesis because none of the current isolates lacked the *ompF* porin.

It is important to note that, among the 13 ESCR strains, only two showed resistance to all tested CEPH, including the fourth-generation CPM and were the only strains that harbored *bla*CTX-M; these were phenotypically non-ESBL-producing and CR. Since *bla*CMY-2 can hydrolyze all β-lactams except the fourth generation CEPH and carbapenems [5,6], the resistance of the two strains to CPM, might be due to the presence of *bla*CTX-M. In this regard, it was reported that resistance of *Salmonella* to CPM is mediated by *bla*CTXM-55, and that this beta-lactamase was shown to be resistant to clavulanic acid and other beta-lactamase inhibitors [36].

Our findings imply an extensive use of CEPH in chickens, which could exert selective pressure for resistance to extended-spectrum CEPH and carbapenem drugs [37].

To the best of our knowledge, the current study is the first to show the presence of ESCR and CR *Salmonella* in chicken giblets in Egypt. In this regard, Salmonella was previously isolated from chicken giblets in Egypt but was not tested for ESBL or CR [38]. In addition, Ceftriaxone and Cefotaxime-resistant *Salmonella* typhimurium was isolated from chicken meat; the isolates carried *bla*TEM, while *bla*SHV, *bla*OXA, and *bla*CMY-2 were absent [39].

Examination of the seven water tanks used for cleaning the chicken carcasses resulted in the isolation of two ESCR, phenotypically non-ESBL-producing and CR *Salmonella* strains, carrying both ESBL and *bla*KPC genes, with one strain carrying *bla*CMY-2. Like in water, two ESCR non-ESBL and CR *Salmonella* strains were isolated from hand swab samples of 16 workers handling the chicken carcasses. However, the serotypes of *Salmonella* isolated from giblets, water, and humans were different; the resistance phenotype and genotype were similar. This suggests a possible transfer of plasmids carrying ESBL genes, *bla*CMY-2, and *bla*KPC among different serotypes of *Salmonella*, as well as between humans and animals.

## Conclusion

ESCR *S. enterica* was isolated from retail chicken giblets; the isolates were resistant to more than one CEPH and harbored ESBL and *bla*CMY-2 genes. A major number of these strains were CR, some harbored *bla*KPC, and the others were negative to the three examined carbapenemase genes. ESCR CR *S. enterica* carrying ESBL, *bla*CMY-2, and *bla*KPC genes was also recovered from water tanks and the hands of workers at the retail poultry shops. Given the possibility of horizontal transfer of AR genes among different types of bacteria in animals and humans [40], the current findings pose a public health danger. In Egypt, food shops are regularly inspected for food quality and adherence to hygienic measures. Shops breaking biosafety rules can be closed down. However, the present study highlights the need for a controlled assessment of the use of antibiotics in the veterinary field and urges for regular screening of AR bacteria in the food chain, contact people, and the surrounding environment. This should be done in parallel with an increase in public awareness of the use of antibiotics in humans.

## Data Availability

The datasets generated during and/or analyzed during the study is available from the corresponding author on reasonable request.

## Authors’ Contributions

All authors conceived and designed the study. FA: Conducted experiments. FA, EH, and KAA: Analyzed the data. FA, EH, and MAS: Drafted and revised the manuscript. All authors read and approved the final manuscript.

## References

[1] Centers for Disease Control and Prevention (2020). Antibiotic/AMR.

[2] Clinical Laboratory Standards Institute (2020). Performance Standards for Antimicrobial Susceptibility Testing, M100.

[3] Bennett J.E, Bennett J.E, Dolin R, Blaser M.J (2020). Cephalosporins. Mandell, Douglas, and Bennett's Principles and Practice of Infectious Diseases.

[4] Pfeifer Y, Cullik A, Witte W (2010). Resistance to cephalosporins and carbapenems in Gram-negative bacterial pathogens. Int. J. Med. Microbiol.

[5] Meini S, Tascini C, Cei M, Sozio E, Rossolini G.M (2019). AmpC ?-lactamase-producing Enterobacterales:What a clinician should know. Infection.

[6] Wilson H, Török M.E (2018). Extended-spectrum ?-lactamase-producing and carbapenemase-producing *Enterobacteriaceae*. Microb. Genom.

[7] Bush K, Jacoby G.A (2010). Updated functional classification of beta-lactamases. Antimicrob. Agents Chemother.

[8] Folster J.P, Pecic G, McCullough A, Rickert R, Whichard J.M (2011). Characterization of bla (CMY)-encoding plasmids among *Salmonella* isolated in the United States in 2007. Foodborne Pathog. Dis.

[9] Rensing K.L, Abdallah H.M, Koek A, Elmowalid G.A, Vandenbroucke-Grauls C.M.J, Al Naiemi N, van Dijk K (2019). Prevalence of plasmid-mediated AmpC in *Enterobacteriaceae* isolated from humans and from retail meat in Zagazig, Egypt. Antimicrob. Resist. Infect. Control.

[10] Jeon H.Y, Kim Y.B, Lim S.K, Lee Y.J, Seo K.W (2019). Characteristics of cephalosporin-resistant Salmonella isolates from poultry in Korea, 2010-2017. Poult. Sci.

[11] Logan L.K, Weinstein R.A (2017). The epidemiology of carbapenem-resistant *Enterobacteriaceae*:The impact and evolution of a global Menace. J. Infect. Dis.

[12] Van Boxtel R, Wattel A.A, Arenas J, Goessens W.H, Tommassen J (2016). Acquisition of carbapenem resistance by plasmid-encoded-AmpC-expressing *Escherichia coli*. Antimicrob. Agents Chemother.

[13] Koga V.L, Maluta R.P, da Silveira W.D, Ribeiro R.A, Hungria M, Vespero E.C, Nakazato G, Kobayashi R.K.T (2019). Characterization of CMY-2-type beta-lactamase-producing *Escherichia coli* isolated from chicken carcasses and human infection in a city of South Brazil. BMC Microbiol.

[14] Dame-Korevaar A, Fischer E, van der Goot J, Stegeman A, Mevius D (2019). Transmission routes of ESBL/pAmpC producing *Bacteria* in the broiler production pyramid, a literature review. Prev. Vet. Med.

[15] Fernández J, Guerra B, Rodicio M.R (2018). Resistance to carbapenems in Non-Typhoidal *Salmonella enterica* serovars from humans, animals and food. Vet. Sci.

[16] Huang J, Wang M, Ding H, Ye M, Hu F, Guo Q, Xu X, Wang M (2013). New Delhi metallo-?-lactamase-1 in carbapenem-resistant *Salmonella strain*, China. Emerg. Infect. Dis.

[17] Miriagou V, Tzouvelekis L.S, Rossiter S, Tzelepi E, Angulo F.J, Whichard J.M (2003). Imipenem resistance in a Salmonella clinical strain due to plasmid-mediated class A carbapenemase KPC-2. Antimicrob. Agents Chemother.

[18] Wang W, Baloch Z, Peng Z, Hu Y, Xu J, Fanning S, Li F (2017). Genomic characterization of a large plasmid containing a *bla*NDM_-1_ gene carried on *Salmonella enterica* serovar Indiana C629 isolate from China. BMC Infect. Dis.

[19] Liu J, Zhu Y, Jay-Russell M, Lemay D.G, Mills D.A (2020). Reservoirs of antimicrobial resistance genes in retail raw milk. Microbiome.

[20] ISO 6579 (1998). Microbiology of Food and Animal Feeding Stuff-Horizontal Method for the Detection of *Salmonella*.

[21] Bezuidenhout C, Mthembu N, Puckree T, Lin J (2002). Microbiological evaluation of the Mhlathuze River, KwaZulu-Natal (RSA). Water SA.

[22] Popoff M.Y (2001). Antigenic formulas of the *Salmonella* serovars.

[23] Reischl U, Pluz M, Ehret W, Wolf H (1994). PCR-based detection of mycobacteria in sputum samples using a simple and reliable DNA extraction protocol. BioTechniques.

[24] Rahn K, De Grandis S.A, Clarke R.C, McEwen S.A, Galán J.E, Ginocchio C, Curtiss R (1992). Amplification of an invA gene sequence of *Salmonella* Typhimurium by polymerase chain reaction as a specific method of detection of *Salmonella*. Mol. Cell Probes.

[25] Murugkar H.V, Rahman H, Dutta P.K (2003). Distribution of virulence genes in *Salmonella* serovars isolated from man and animals. Indian J. Med. Res.

[26] Kataria J, Kumar A, Rajagunalan S, Jonathan L, Agarwal R (2013). Detection of OmpA gene by PCR for specific detection of *Salmonella* serovars. Vet. World.

[27] Tatavarthy A, Cannons A (2010). Real-time PCR detection of *Salmonella* species using a novel target:The outer membrane porin F gene (ompF). Lett. Appl. Microbiol.

[28] Djeffal S, Bakour S, Mamache B, Elgroud R, Agabou A, Chabou S, Hierche S, Bouaziz O, Rahal K, Rolain J.M (2017). Prevalence and clonal relationship of ESBL-producing *Salmonella* strains from humans and poultry in Northeastern Algeria. BMC Vet. Res.

[29] Kim J, Jeon S, Rhie H, Lee B, Park M, Lee H, Lee J, Seonghan K (2009). Rapid detection of extended-spectrum ?-lactamase (ESBL) for *Enterobacteriaceae* by use of a multiplex PCR-based method. Infect. Chemother.

[30] Li B, Yi Y, Wang Q, Woo P.C.Y, Tan L, Jing H, Gao G.F, Liu C.H (2012). Analysis of drug resistance determinants in *Klebsiella pneumoniae* isolates from a tertiary-care hospital in Beijing, China. PLoS One.

[31] Ammar A.M, Abdeen E.E, Abo-Shama U.H, Fekry E, Kotb Elmahallawy E (2019). Molecular characterization of virulence and antibiotic resistance genes among *Salmonella* serovars isolated from broilers in Egypt. Lett. Appl. Microbiol.

[32] Sabry M, Abdel-Moein K, Abdel-Kader F, Hamza E (2020). Extended-spectrum beta-lactamase-producing Salmonella serovars among healthy and diseased chickens and its public health implication. J. Glob. Antimicrob. Resist.

[33] Bajpai T, Pandey M, Varma M, Bhatambare G.S (2018). Prevalence of TEM, SHV, and CTX-M beta-lactamase genes in the urinary isolates of a tertiary care hospital. Avicenna J. Med.

[34] Bortolami A, Zendri F, Maciuca E.I, Wattret A, Ellis C, Schmidt V, Pinchbeck G, Timofte D (2019). Diversity, virulence, and clinical significance of extended-spectrum ?-lactamase-and pAmpC-producing *Escherichia coli* from companion animals. Front. Microbiol.

[35] Hamza E, Dorgham S.M, Hamza D.A (2016). Carbapenemase-producing *Klebsiella pneumoniae* in broiler poultry farming in Egypt. J. Glob. Antimicrob. Resist.

[36] Fu Y, Xu X, Zhang L, Xiong Z, Ma Y, Wei Y, Chen Z, Bai J, Liao M, Zhang J (2020). Fourth generation cephalosporin resistance among *Salmonella enterica* serovar enteritidis isolates in Shanghai, China conferred by *bla*CTX-M-55 Harboring plasmids. Front. Microbiol.

[37] Baron S, Diene S, Rolain J (2018). Human microbiomes and antibiotic resistance. Hum. Microb. J.

[38] Abd-ElGhany S.M, Sallam K.I, Abd-Elkhalek A, Tamura T (2015). Occurrence, genetic characterization, and antimicrobial resistance of *Salmonella* isolated from chicken meat and giblets. Epidemiol. Infect.

[39] Ahmed H.A, El-Hofy F.I, Shafik S.M, Abdelrahman M.A, Elsaid G.A (2016). Characterization of virulence-associated genes, antimicrobial resistance genes, and class 1 integrons in *Salmonella enterica* serovar Typhimurium isolates from chicken meat and humans in Egypt. Foodborne Pathog. Dis.

[40] González-Sanz R, Herrera-León S, de la Fuente M, Arroyo M, Echeita M.A (2009). Emergence of extended-spectrum beta-lactamases and AmpC-type beta-lactamases in human *Salmonella* isolated in Spain from 2001 to 2005. J. Antimicrob. Chemother.

